# Supplementary material for: Spatial metabolomics reveals the role of penicillic acid in cheese-rind microbiome disruption by a spoilage fungus
Source: mSystems. 2026 Jan 12;11(2):e01305-25. doi: 10.1128/msystems.01305-25 (PMC12911348; doi:10.1128/msystems.01305-25)
Supplement: Supplemental figures — Figures S1 to S8 and legends for Tables S1 to S3. [file msystems.01305-25-s0001.pdf]

Supporting Information for:

Spatial Metabolomics Reveals the Role of Penicillic Acid in Cheese Rind Microbiome Disruption by a Spoilage Fungus - Supplementary Material

Authors: Carlismari O. Grundmann [a], Christopher Tomo [b], Julia Hershelman [b], Benjamin E. Wolfe [b]*, Laura M. Sanchez [a]*

[a] Department of Chemistry and Biochemistry - University of California Santa Cruz, Santa Cruz, California, USA

[b] Department of Biology, Tufts University, Medford, Massachusetts, USA

* Corresponding authors: lmsanche@ucsc.edu, benjamin.Wolfe@tufts.edu

Table of Contents

Legends for Tables S1, S2, and S3………………………………………………………………… S2
Figure S1. Comparative analysis of biosynthetic gene clusters in *A. westerdijkiae*……. S3
Figure S2. GNPS mirror match plot for penicillic acid…………...……………………………. S4
Figure S3. GNPS mirror match plot for circumdatin A……………………………………….... S5
Figure S4. GNPS mirror match plot for circumdatin F…………………………………….. S6
Figure S5. Spatial distribution of mycotoxins in *A. westerdijkiae* and *Staphylococcus*... S7
Figure S6. Calibration curve for penicillic acid quantification……………………….……….. S8
Figure S7. Bromocresol purple calibration and pH estimation in interaction plates……………. S9
Figure S8. Multivariate analysis and metabolite annotation…..…………………………….. S10

Table S1. Functional pathways enriched with differentially expressed genes of *S. equorum* grown in monoculture to co-culture growth with *A. westerdijkiae* at two time points. Full list of KEGG functional pathways enriched with differentially expressed genes when *S. equorum* was grown in co-culture with *A. westerdijkiae* for 96 h. Analysis was conducted using the KOBAS-i gene-list enrichment tool (version 7.0).

Table S2. Predicted BGCs identified in *A. westerdijkiae* genome sequence via antiSMASH. 78 BGCs were predicted in the fungal genome and for each region, the table reports the region identifier, BGC type (e.g., NRPS, PKS, RiPP-like, etc.), genomic coordinates (start and end positions), and the most similar known compound predicted. The color annotations correspond to different levels of annotation confidence and integration with metabolomic data. Regions highlighted in red indicate BGCs with no known compound predicted. Orange regions represent BGCs with putative compound matches that show less than 50% similarity to known clusters. Green regions correspond to BGCs with predicted compound similarity equal to or greater than 50%, which were subsequently cross-referenced with MSI data.

Table S3. Complete dataset for bromocresol purple pH calibration curve and semi-quantitative analysis of pH gradients during *A. westerdijkiae* interactions. The table reports (i) the colorimetric measurements (Blue/Red ratios) of pH reference standards, (ii) Blue/Red ratios obtained from ROI measurements in monoculture and co-culture plates, and (iii) the estimated pH values for each ROI derived from the curve. These data were used to generate Fig. S7 and evaluate potential pH-driven effects during fungal–bacterial interactions.

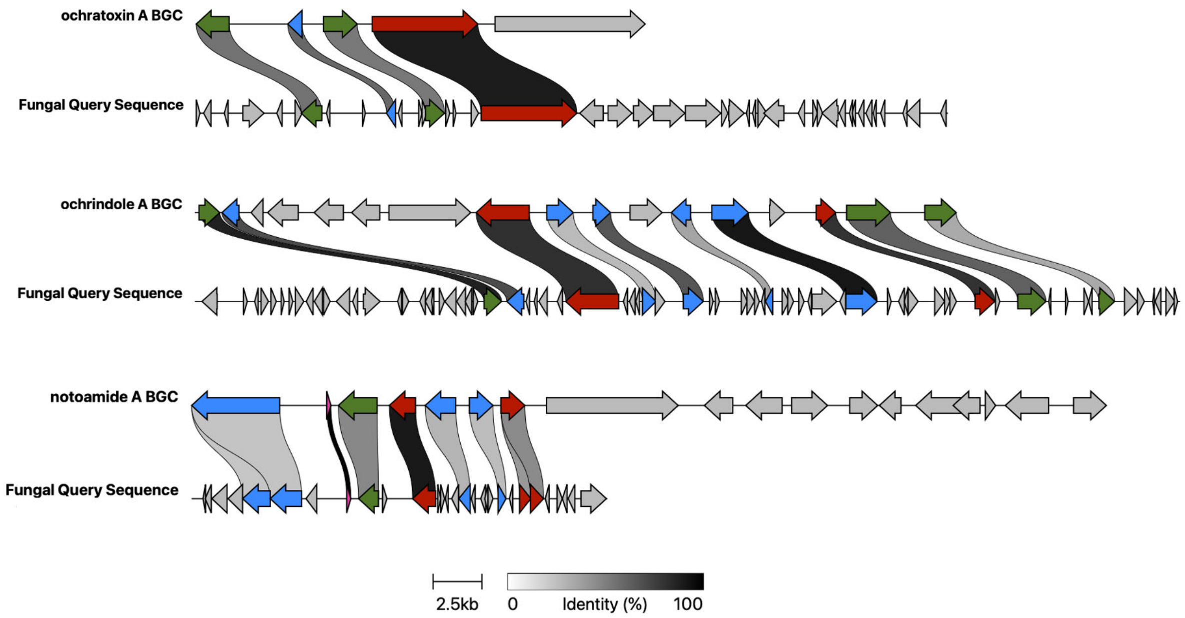

Figure S1. Comparative analysis of biosynthetic gene clusters (BGCs) in *A. westerdijkiae* genome with known clusters for ochratoxin A, ochrindole A, and notoamide A. Clusters were predicted using antiSMASH and visualized using Clinker. Arrows represent genes; colored links indicate homologous genes with amino acid identity scaled from 0% (light gray) to 100% (black). Core biosynthetic, tailoring, regulatory, and additional genes are color-coded as indicated.

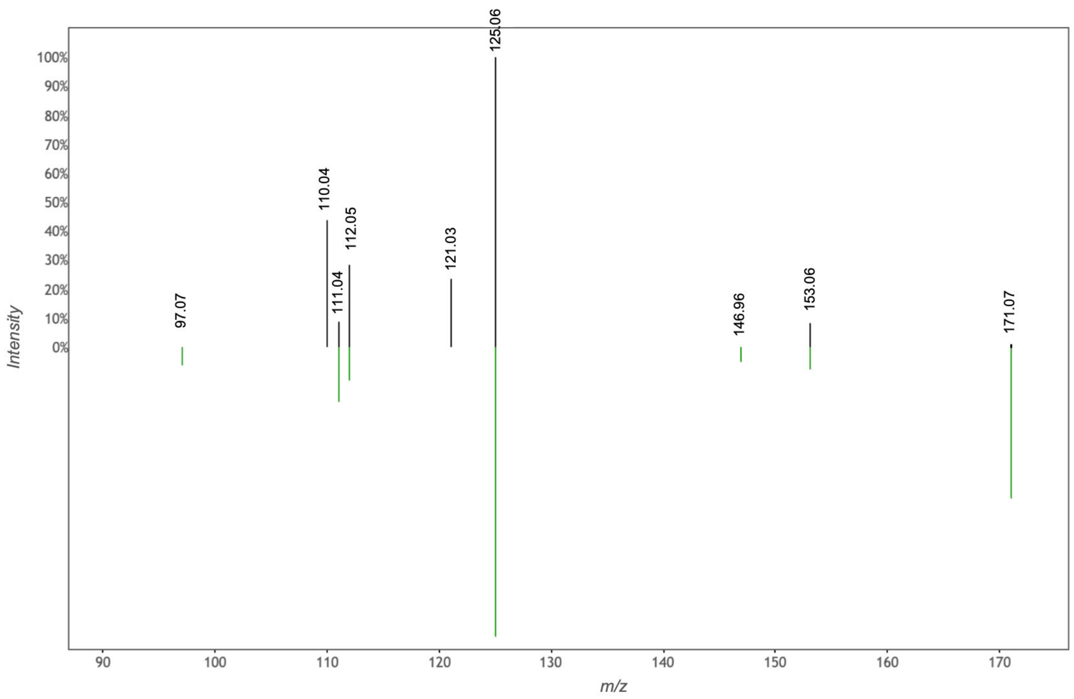

Figure S2. GNPS mirror match plot for penicillic acid **(6)**. Green peaks represent the MS/MS library spectrum, while black peaks correspond to the MS/MS spectrum acquired from microbial samples. The comparison yielded 4 shared peaks and a cosine score of 0.77. This feature, corresponding to the ion with *m/z* 171.06, was detected in *A. westerdijkiae* monocultures and in *A. westerdijkiae* × *Brachybacterium* co-culture samples.

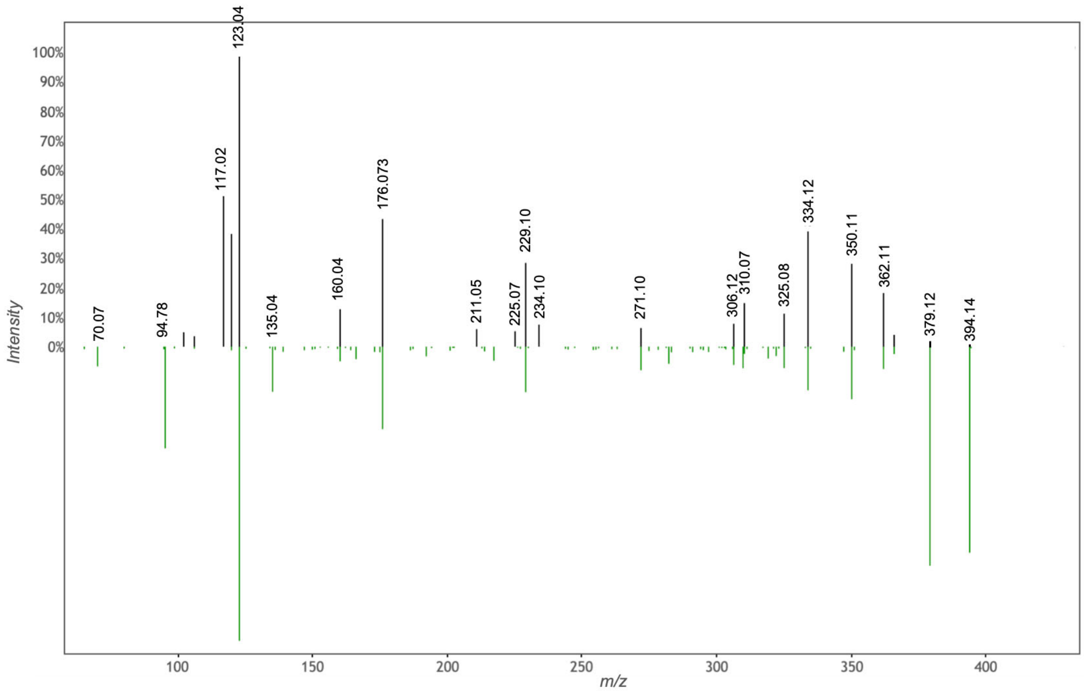

Figure S3. GNPS mirror match plot for circumdatin A **(7)**. Green peaks represent the MS/MS library spectrum, while black peaks correspond to the MS/MS spectrum acquired from microbial samples. The comparison yielded 11 shared peaks and a cosine score of 0.72. This feature, corresponding to the ion with *m/z* 394.14, was detected in *A. westerdijkiae* monocultures and in *A. westerdijkiae* × *Brachybacterium* co-culture samples.

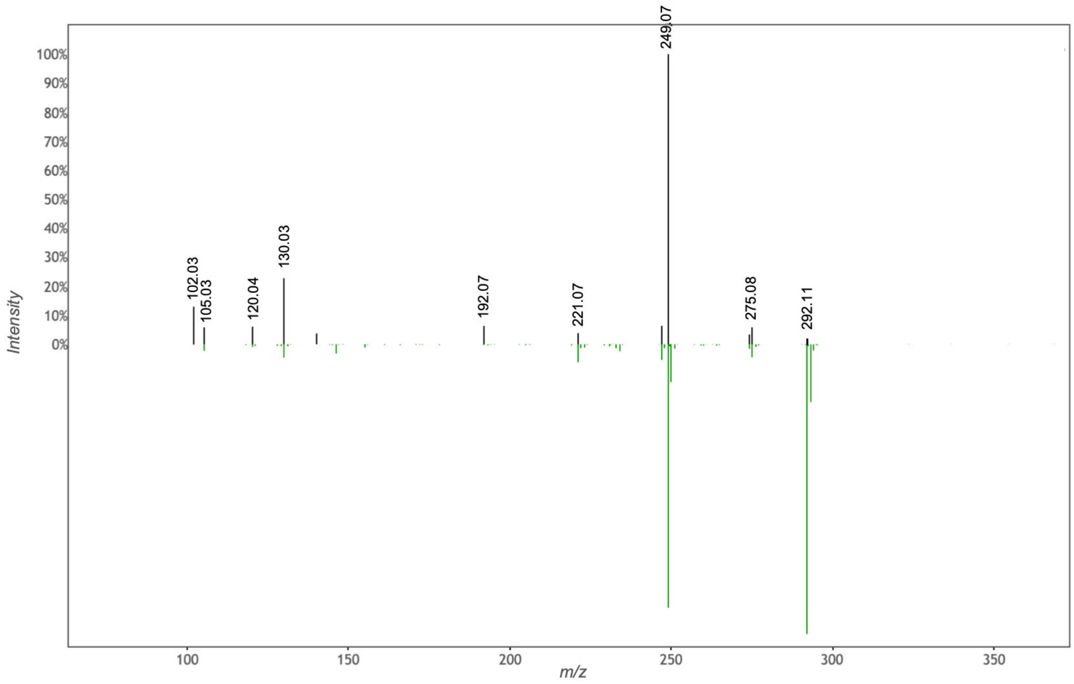

Figure S4. GNPS mirror match plot for circumdatin F **(9)**. Green peaks represent the MS/MS library spectrum, while black peaks correspond to the MS/MS spectrum acquired from microbial samples. The comparison yielded 8 shared peaks and a cosine score of 0.83. This feature, corresponding to the ion with *m/z* 292.11, was detected in *A. westerdijkiae* monocultures and in *A. westerdijkiae* × *Brachybacterium* co-culture samples.

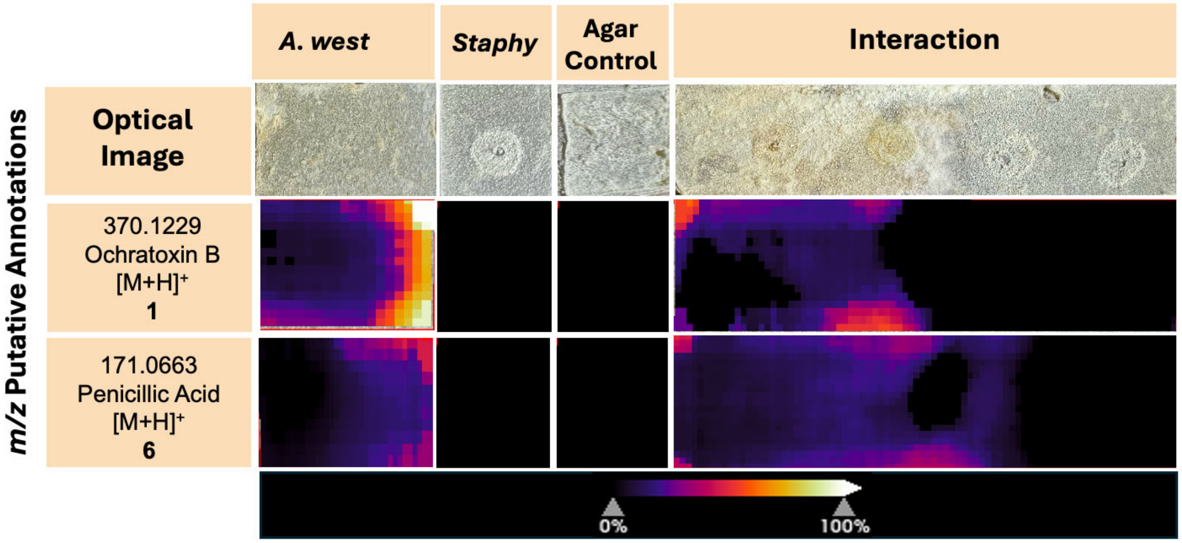

Figure S5. Spatial distribution of mycotoxins in mono- and co-cultures of *A. westerdijkiae* and *Staphylococcus*. Optical images and MALDI imaging mass spectrometry heat maps show spatial localization of key mycotoxins ochratoxin B [M+H]+, *m/z* 370.1229 and penicillic acid [M+H]+, *m/z* 171.0663 detected in the samples. The abundance of *m/z* signals were normalized to total ion count intensities across the following conditions: *A. westerdijkiae* monoculture, *Staphylococcus* monoculture, agar control, and the co-culture interaction. The mass accuracy error is provided for each putative annotation.

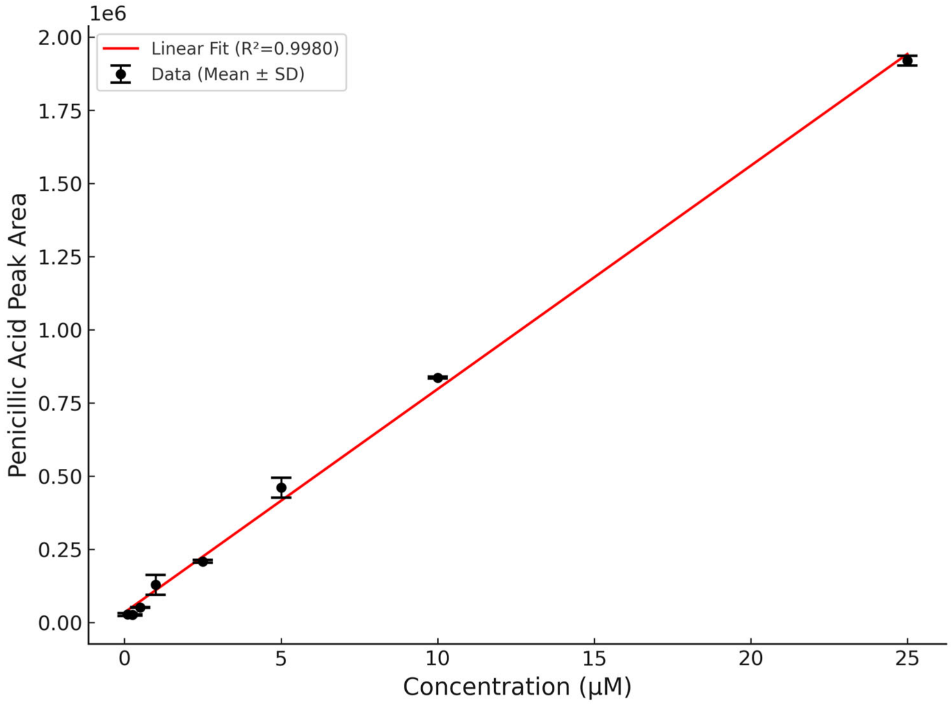

Figure S6. Calibration curve for penicillic acid quantification. The graph shows the mean peak area (± standard deviation) of penicillic acid detected by LC-MS at increasing concentrations (0.1, 0.25, 0.5, 1, 2.5, 5, 10, and 25 μM). Each point represents the mean of three replicates, with error bars indicating standard deviation. A linear regression (red line) was fitted to the data, resulting in the equation $y = 76297.52 \cdot x + 34868.49$ and an R^2 value of 0.9980.

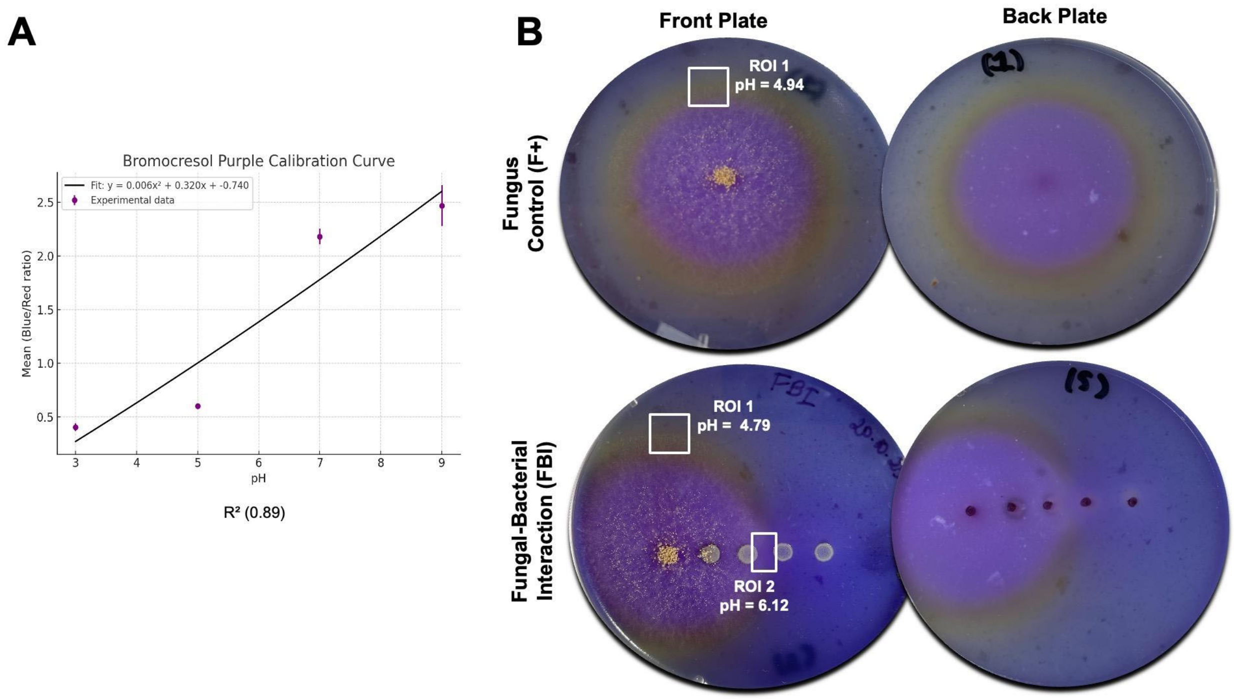

Figure S7. Bromocresol purple calibration and pH estimation in interaction plates.**(A)** Color-pH calibration curve generated from plates prepared at pH 3, 5, 7, and 9 with 0.02 g/L bromocresol purple in 2.5% CCA, imaged under standardized lighting and quantified in ImageJ with Blue/Red intensity ratios. **(B)** Representative fungal control (F+) and fungal–bacterial interaction (FBI) plates (front and back) showing ROIs used for analysis and its respective estimated pH values, indicating consistent acidification by *A. westerdijkiae* and a mildly acidic/near-neutral transition zone toward bacterial inocula.

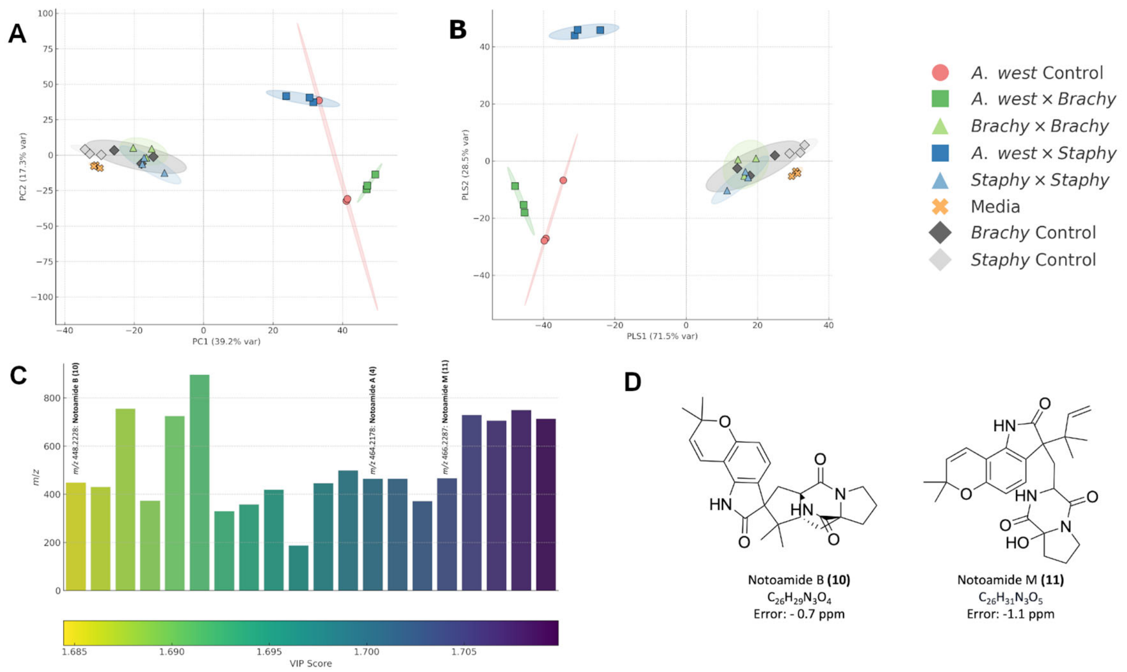

Figure S8. Multivariate analysis and metabolite annotation reveal differential production of notoamides during fungal-bacterial interactions. **(A)** Principal Component Analysis (PCA) score plot based on TIC-normalized metabolomics data, showing clear separation between experimental groups along the first two components (PC1: 39.2%, PC2: 17.3% of total variance). Ellipses represent 2.5 standard deviations from the group centroid. **(B)** Partial Least Squares Discriminant Analysis (PLS-DA) score plot showing supervised group separation (PLS1: 71.5%, PLS2: 28.5% of total variance).**(C)** Top 20 discriminant *m/z* features identified by VIP scores derived from the PLS-DA model. The color gradient reflects VIP values, and annotated features putatively identified as notoamides are highlighted. **(D)** Chemical structures of selected putatively annotated metabolites enriched in the fungal interaction with *S. equorum.*
